# COVID-19 during pregnancy should we really worry from vertical transmission or rather from fetal hypoxia and placental insufficiency? A systematic review

**DOI:** 10.1186/s43054-021-00056-0

**Published:** 2021-04-12

**Authors:** Antoine AbdelMassih, Raghda Fouda, Rana Essam, Alhussein Negm, Dalia Khalil, Dalia Habib, George Afdal, Habiba-Allah Ismail, Hadeer Aly, Ibrahim Genedy, Layla El Qadi, Leena Makki, Maha Shulqamy, Maram Hanafy, Marian AbdelMassih, Marina Ibrahim, Mohamed Ebaid, Monica Ibrahim, Nadine El-Husseiny, Nirvana Ashraf, Noura Shebl, Rahma Menshawey, Rama Darwish, Rana ElShahawi, Rana Ramadan, Sadra Albala, Salwa Imran, Sama Ahmed, Samer Khaldi, Sara Abohashish, Stavro Paulo, Yasmin Omar, Mourad Alfy Tadros

**Affiliations:** 1 Pediatric Cardiology Unit, Pediatrics Department, Faculty of Medicine, Cairo University Children Hospital, Cairo University, Kasr Al Ainy Street, Cairo, 12411 Egypt; 2 Pediatric Cardio-Oncology Clinic, Children Cancer Hospital of Egypt, Cairo, Egypt; 3 University of Irvine California, Irvine, USA; 4 Clinical and Chemical Pathology Department, Faculty of Medicine, Cairo University, Cairo, Egypt; 5 Neonatology unit, Pediatrics’ Department, Faculty of Medicine, Cairo University, Cairo, Egypt; 6 Student and Internship research program (Research Accessibility Team), Faculty of Medicine, Cairo University, Cairo, Egypt; 7 Residents’ Training program, Faculty of Medicine, Cairo University, Cairo, Egypt; 8 Pediatrics, 6th October University, Cairo, Egypt; 9 Faculty of Dentistry, Cairo University, Cairo, Egypt; 10 Pixagon Graphic Design Agency, Cairo, Egypt; 11 Military Medical College, Cairo, Egypt; 12 Royal College of Pediatrics and Child Health, London, UK

**Keywords:** COVID-19, Neonatal outcome, Placental infarctions, Fetal hypoxia, Vertical transmission

## Abstract

**Background:**

COVID-19 is the largest outbreak to strike humanity. The wide scale of fatalities and morbidities lead to a concurrent pandemic of uncertainty in scientific evidence. Conflicting evidences are released on daily basis about the neonatal outcomes of COVID-19-positive mothers. The aim of this study was to use the relevant case reports and series to determine the percentage of newborns who test positive for COVID-19 who are born to COVID-19-positive mothers. Secondary outcomes included examining laboratory abnormalities among COVID-19-positive neonates, and any depicted placental abnormalities in COVID-19-positive mothers. For this purpose, systematic review was performed on all studies reporting primary data on fetus-mother pairs with COVID-19. Data bases were searched for studies that met our inclusion and exclusion criteria.

**Results:**

Final screening revealed 67 studies, from which the primary data of 1787 COVID-19 mothers were identified and had their pregnancy outcome analyzed. Only 2.8% of infants born to COVID-19-positive mothers tested positive, and this finding is identical to percentages reported in former Coronaviridae outbreaks, whereas 20% manifested with intrauterine hypoxia alongside placental abnormalities suggestive of heavy placental vaso-occlusive involvement.

**Conclusions:**

These findings suggest that while vertical transmission is unlikely, there appears to be an underlying risk of placental insufficiency due to the prothrombotic tendency observed in COVID-19 infection. Guidelines for proper prophylactic anticoagulation in COVID-positive mothers need to be established.

## Background

COVID-19 (coronavirus disease 2019), which has been declared a pandemic in March 2020, has caused an unprecedented uncertainty within the scientific community. Contradictory scientific evidences are released almost every day, on every aspect of the pandemic from its pathogenesis, to the methods of transmission, and to the possible compassionate use of medications to combat it. Transplacental transmission of COVID-19 is one of the topics that have raised conflicting evidences across the globe. The dilemma about transplacental transmission of Coronaviridae is not exclusive to the current outbreak. To our knowledge, nine studies [1–9] from SARS-1 (Severe Acute Respiratory syndrome) and HKCoV (Hong Kong Coronavirus) and MERS (Middle East Respiratory syndrome) outbreaks were reported, ranging from case reports to retrospective case reviews, comprising 71 mother-infant pairs. Table 1 summarizes the findings of the nine studies. Two cases only have shown vertical transmission, a remarkable finding was the strong evidence in those reports of intrauterine fetal hypoxia possibly due to placental damage or even direct evidence of placental infarctions. Gagneur et al. [6] reported two cases of still birth that was preceded by fetal heart deceleration, whereas Wong et al. [3] and Jeong et al. [1] demonstrated placental infarction in three cases. Analysis of placental outcomes was largely lacking in the studies performed in the previous outbreaks; however, all studies that mentioned the presence of placental vascular compromise namely Wong et al. [3] and Jeong et al. [1] excluded the presence of any maternal co-morbidity that can cause such finding (Table 1). The latter finding might signify that CoV are mainly implicated in the thrombotic injury observed in such case reports. The vascular tropism of COVID-19 has recently gained so much interest, and many of its multi-organ manifestations has been attributed to its endothelial tropism. Such endothelial tropism is accounted for by the high load of Angiotensin Converting Enzyme 2 (ACE2) and Furin [10, 11], which are important viral checkpoints, in the endothelium. The placenta is a vascular organ, in which Furin plays an important role in its differentiation; moreover, ACE2 and angiotensin 1-7 are heavily expressed in it, making the placenta an important target for the vascular tropic effect of COVID-19. As mentioned earlier, the conflicting evidence regarding vertical transmission of COVID-19 and the effect of maternal COVID-19 on newborns and their placenta, render systematic review of the clustered cases available of utmost importance to build stronger evidence for the neonatal outcomes of COVID-19. The primary outcome parameter of this systematic review is the percentage of newborns testing positive to COVID-19 mothers, while secondary outcome parameters included the assessment of laboratory abnormalities among COVID-19 newborns, and the placental abnormalities encountered in COVID-19 mothers.

**Table 1:** Reported cases of vertical transmission, clinical manifestations and placental abnormalities in SARS-1, HKCoV, and MERS

Paper	Jeong et al [1]	Payne et al [2]	Wong et al [3]	Yudin et al [4]	Stockman et al [5]	Gagneur et al [6]	Li et al [7]	Shek et al [8]	Robertson et al [9]	Totals
Outbreak	MERS	MERS	SARS-1	SARS-1	MERS	HKCoV/SARS	SARS-1	SARS-1	SARS-1	
Number of studied mother-infant pairs	1	1	12	1	2	7	41	5	1	71
Number of neonates with vertical transmission	0	0	0	0	0	2	0	0	0	2/71 (2.8%)
Reported complications in neonates(whether with positive or negative swabs)	0	1: Still birth	2: NEC 1: RDS	0	0	2: Still birth with fetal deceleration	0	2: NEC	0	RDS: 1/71 (1.4%) Still birth: Mar-71 − 4.20% NEC: 4/71 (5.6%)
Reported placental abnormalities	2: Placental infarction	Not reported	1: Placental infarction	Not reported	Placenta negative for SARS-CoV, no pathological examination	Not reported	Not reported	Not reported	Not reported	3/25 (12%)
Maternal co-morbidities	None	None	None	None	Gestational diabetes in the third trimester	One mother developed eclampsia	None	None	None	

*Abbreviations*: *HKCoV* Hong Kong Coronaviridae, *MERS* Middle East Respiratory Syndrome, *NEC* necrotizing enterocolitis, *RDS* respiratory distress syndrome, *SARS* severe acute respiratory syndrome

## Main text

### Methods

This systematic review has been conducted in agreement with the guidelines of the PRISMA Statement (Preferred Reporting Items for Systematic Reviews and Meta-Analysis) [12].

#### Data search

A computer run has been performed in EMBASE, MEDLINE, and the Cochrane Central Register (From 1 November 2019 to 1 of August 2020). The following terms were included in the search: “COVID-19” OR “SARS-CoV-2” (Severe Acute Respiratory syndrome Coronaviridae 2) AND “Pregnancy” AND “Perinatal”.

#### Study selection criteria

Population: Pregnant women

Intervention: COVID-19

Comparison: No comparison has been a purpose of the study

Outcome: Neonatal infection by COVID-19, placental abnormalities, laboratory abnormalities in the newborn.

Observational epidemiological studies and case reports addressing the clinical conditions of mother–fetus pairs. Primary data of patients over 18 years old were considered eligible. Manuscripts that contained only data from pregnant women, or only fetuses, or that did not address the period of delivery, such as puerperium, were disregarded. All data from eligible studies were extracted by 2 independent investigators according to a standard protocol.

### Statistical analysis

**Fig. 1:**
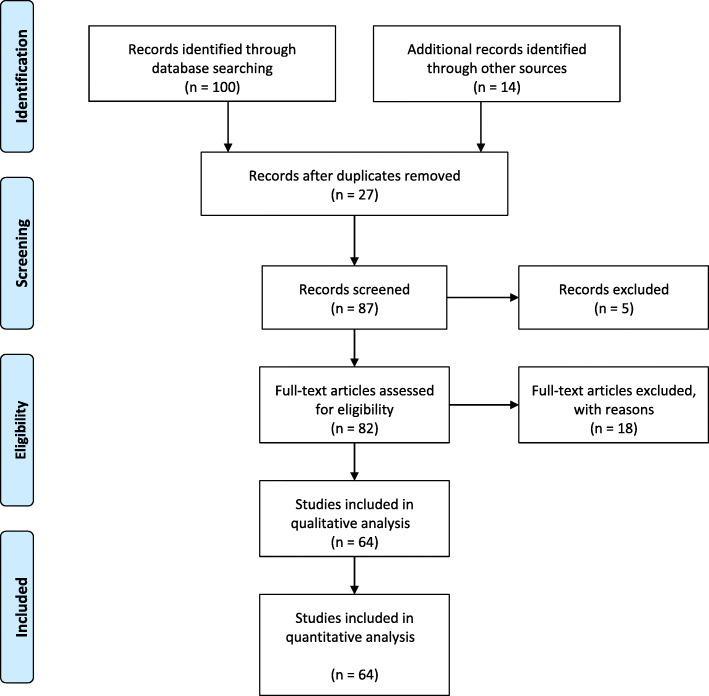
PRISMA flowchart for the process of study selection. Abbreviations: Preferred Reporting Items for Systematic Reviews and Meta-Analyses

Each of the maternal manifestations, neonatal manifestations, placental microscopic and macroscopic changes, and laboratory changes in COVID-19-positive newborns was quantified and expressed as number (*n*) and percentages. Cases where evidence of placental thrombotic process have been detected and in which maternal co-morbidities were mentioned (whether ruled out or confirmed/*n* = 17) have been analyzed using receiver operating characteristic (ROC) analysis, and Fig. 1 is a ROC curve showing absence of possible relationship between maternal co-morbidities and placental thrombotic process with a *P* value of 0.6.

## Results

The literature search identified initially 114 studies, of which 50 studies were excluded. Twenty three were excluded as they did not tackle the primary outcome parameter of the study; while 27 studies were excluded due to repetition. Total number of studies included was 64 studies, comprising 1787 Mother-infant pair [13–76]. (PRISMA flow chart illustrated in Fig. 1 carries more details on the process of selecting the analyzed studies).

The studies carried out in 15 countries are listed in Table 2 alongside their main outcome parameters. The number of COVID-19-positive mothers is 1787 and the number of infants testing positive 45. There was a total of 19 fetal/neonatal deaths reported while only 72 placentae got examined.

**Table 2:** Summary of data in the included studies of vertical transmission of COVID-19

Reference number in the text	Reference	Type of the study	Number of mothers	Number of positive neonates	Number of fetal/neonatal deaths	Cause of fetal death	Placenta examined	Country of origin
[13]	Khan, S. *et al.*	Case series	17	0	0		0	China
[14]	Hantoushzadeh, S. *et al.*	Case series	9	0	3	Intrauterine fetal death with 1 case of non-reassuring cardiotocography	0	Iran
[15]	Chen, S. *et al.*	Case series	5	0	0		0	China
[16]	Chen, R. *et al.*	Retrospective study	17	0	0		0	China
[17]	Ferrazzi, E. *et al.*	Retrospective study	42	1	0		0	Italy
[18]	Dashraath, P. *et al.*	Retrospective study	55	0	2	Intrauterine fetal death with preceding intrauterine growth restriction	0	China
[19]	Baud, D. *et al.*	Case Report	1	0	0		1	Switzerland
[20]	Dong, L. *et al.*	Case Report	1	1	0		0	China
[21]	González Romero, D., et al	Case Report	1	0	0		0	Spain
[22]	Breslin, N. *et al.*	Retrospective study	43	0	0		0	USA
[23]	Alzamora, M. C. *et al.*	Case Report	1	1	0		0	Peru
[24]	Chen, H. *et al.*	Case series	9	0	0		0	China
[25]	Qiancheng, X. *et al.*	Retrospective study	82	0	0		0	China
[26]	Liu, Y., et al	Retrospective study	13	0	0		0	China
[27]	Liao, X., et al	Case Report	1	0	0		0	China
[28]	Yu, N. *et al.*	Case Report	1	1	0		0	China
[29]	Kirtsman, M. *et al.*	Case series	4	0	0		0	Canada
[30]	Kang, X. *et al.*	Case Report	1	0	0		0	China
[31]	Buonsenso, D. *et al.*	Case series	4	0	0		0	China
[32]	Lu, D. *et al.*	Case Report	1	0	0		0	China
[33]	Khan, S. et al.	Case series	3	0	0		0	China
[34]	Kalafat, E. *et al.*	Case Report	1	0	0		1	Turkey
[35]	Karami, P. *et al.*	Case Report	1	0	0		1	Iran
[36]	Nie, R. *et al.*	Retrospective study	33	1	0		0	China
[37]	Lowe, B. Et al	Case Report	1	0	0		0	Australia
[38]	Chen, S. *et al.*	Case series	3	0	0		3	China
[39]	Li, Y. *et al.*	Case series	1	0	0		0	China
[40]	Fan, C. *et al.*	Case series	2	0	0		2	China
[41]	Zambrano, L. I. *et al.*	Case Report	1	0	0		0	Honduras
[42]	Iqbal, S. N. *et al.*	Case Report	1	0	0		0	China
[43]	Wang, X. *et al.*	Case Report	1	0	0		1	China
[44]	Xiong, X. *et al.*	Case Report	1	0	0		0	China
[45]	Lee, D. H. *et al.*	Case Report	1	0	0		0	China
[46]	Yue, L. *et al.*	Case Report	14	0	0		0	China
[47]	Liu, W. *et al.*	Retrospective study	19	0	0		0	China
[48]	Shi, H. *et al.*	Retrospective study	81	0	0		0	China
[49]	Liao, J. *et al.*	Case Report	1	0	0		1	China
[50]	Yin, M. *et al.*	Retrospective study	16	0	0		16	China
[51]	Li, N. *et al.*	Case-Control	34	0	0		0	China
[52]	Shanes, E. D. *et al.*	Retrospective study	16	0	7	IUGR	16	USA
[53]	Knight Dphil, M. *et al.*	Retrospective study	420	24	5	2 from neonatal pneumonia and 3 IUFD	0	UK
[54]	Govind, A. *et al.*	Case series	9	3	0		0	UK
[55]	Kayem, G. *et al.*	Retrospective study	617	2	1	Not specified	0	France
[56]	Nyholm, S. *et al.*	Case Report	1	1	0		0	Sweden
[57]	Easterlin, M. C., et al	Case Report	1	0	0		0	USA
[58]	Wu, Y. *et al.*	Case series	13	0	0		0	China
[59]	Hong, L. *et al.*	Case Report	1	0	0		0	USA
[60]	Vivanti, A. J. *et al.*	Case Report	1	0	0		1	France
[61]	Salvatori, G. *et al.*	Case series	2	2			0	Italy
[62]	Wu, Y.-T. *et al.*	Retrospective study	29	4			0	China
[63]	Sisman, J. *et al.*	Case Report	1	1	0		1	USA
[64]	Yang, P. *et al.*	Case Report	7	0	0		0	China
[65]	Zheng, T. *et al.*	Case series	2	0	0		0	China
[66]	Wang, S. *et al.*	Case Report	1	0	0		1	China
[67]	Dumpa, V., et al	Case Report	1	1	0		0	China
[68]	Masmejan, S. *et al.*	Case series	13	0	0		13	Switzerland
[69]	Yang, H.,et al	Retrospective study	24	1	0		0	China
[70]	Hillary, H. *et al.*	Case Report	1	0	0		1	USA
[71]	Ferraiolo, A. *et al*	Case Report	1	0	0		1	Italy
[72]	Ng, W. F. *et al.*	Case Series	8	0	0		8	Hong Kong
[73]	Edlow, A. G. et al	Case Series	64	0	2	Placental malperfusion	44	USA
[74]	Zeng, H. *et al.*	Case series	6	0	0		0	China
[75]	Liu, D. *et al.*	Case series	15	0	0		0	China
[76]	Zhu, H. *et al.*	Case series	10	0	1	Multiple organ failure and DIC few hours after birth/Small for gestational age	0	China
Totals	-		1787	45	21		112	-

*Abbreviations*: *COVID-19* Coronavirus Disease 2019, *DIC* Disseminated intravascular coagulation, *IUGR* Intrauterine growth retardation, *IUFD* Intrauterine fetal death, *UK* United Kingdom, *USA* United States of America

These data are further analyzed in Table 3 which summarizes the clinical manifestations of included COVID-19-positive mothers and the subsequent percentage of positive newborns. Out of 1787 mother-infant pairs, only 45 tested positive (2.5%), which is nearly identical to the percentage of neonates affected in the reported case series during the previous three outbreaks caused by Coronaviridae, 2/71 (2.8%) (Table 1). Among COVID-19-positive neonates, 24% were asymptomatic. The commonest array of manifestations among COVID-19-positive neonates was those suggestive of intrauterine hypoxia (20%).

**Table 3:** Gestational age, infection timing, and clinical characteristics of COVID-19-positive mothers and neonates

Maternal manifestations among COVID-19-positive mothers (*n* = 1787) (*n*/%)	Newborn manifestations among COVID-19-positive neonates (*n*/%)	Sample time (*n*/%)	GA in COVID-19-positive neonates (*n*/%)	Trimester of acquiring infection in COVID-19-positive mothers (*n*/%)
***Asymptomatic (219/12%)*** ***Respiratory manifestations:*** -Cough (934/52.2%) -Dyspnoea (417/23.3%) -Respiratory support needed (130/7%) -Expectoration (1/0.05%) -Sore throat (44/2.4%) -Minor symptoms (124/7%) -Critical symptoms (35/2%) -Rhinorrhoea (20/1%) -Chest pain (2/0.1%)) ***GIT manifestations:*** -Diarrhea (78/4.4%) -Abdominal pain (2/0.1%) -Vomiting (41/2.2%) ***CNS manifestations:*** -Limb asthenia (1/0.05%) -Anosmia (180) -Lethargy (76/4.3%) -Dysgeusia (1/0.05%) -Headache (60/3.3%) ***Others:*** - Fever (951/53%) -Myalgia & joint pain (56/3%) -Back pain (2/0.1%) -Tachycardia (1/0.05%) -Tachypnea (1/0.05%)	***Asymptomatic (11/24%)*** ***Respiratory manifestations (8/17%)*** -Pneumonia (4/8%) -Intubated (2/4%) - Ventilated (1/2%) -Cough (1/2%) ***CNS manifestations (3/7%)*** -Irritability (1/2%) -Axial hypertonia and opisthotonus (1/2%) -Neonatal encephalopathy (1/2%) ***Feeding disorders and GERD (7/15%)*** ***Evidence of intrauterine fetal asphyxia: (9/20%)*** -Meconium-stained liquor (3/7%) - Suboptimal cardiotocography: Fetal late heart deceleration/fetal bradycardia (6/14%) -IUGR (3/7%) ***Others: (6/13%)*** -Myocardial dysfunction/cardiogenic shock (2/4%) -Hypoglycaemia (1/2%) -Fever (2/4%) -Diarrhea (1/2%) **Not reported (14/31%)**	***Less than 12 h (19 /42%)*** ***< 24 h and > 12 h (7 /16%)*** **After 24 h (5 /11%)** ***Not reported (14/31%)***	***Not reported*** (26 /57.8%) ***Preterm and IUGR*** ***(3/7%)*** 24 W (1/2.2%) 34 W (1/2.2%) 35 W (1/2.2%) ***Full term*** ***(16/35%)*** 36 W (4/8.9%) 37 W (2/4.4%) 38 W (6 /13.3%) 39 W (2/4.4%) 40 W (2/4.4%)	***1st trimester*** (46/3%) ***2nd trimester*** (273/16%) ***3rd trimester*** (1313/73%) ***Not reported*** (155/8%)
***There is overlap of manifestations***		***Total: 45 COVID-19-positive neonates***		***Total: 1787 COVID-19 mothers***

*Abbreviations*: *COVID-19* Coronaviridae, *CNS* central nervous system, *GA* gestational age, *GERD* gastroesophageal reflux, *IUGR* intrauterine growth retardation, *NB* evidence of fetal hypoxia was defined in the table as IUGR, meconium staining of newborn, and IUGR

Table 4 outlines the placental abnormalities in COVID-19-positive mothers. Placental infarction, an evidence of vascular compromise of the villi, was observed in a significant number of abnormal placentae (64%). Positive swabs retrieved from abnormal placentae accounted for 27% of all abnormal placentae, and 15% of all examined placentae, a percentage lower than that of infarcted placentae. A closer percentage of placental infarctions was observed in placentae examined from the previous outbreak.

**Table 4:** Placental abnormalities in placentae of COVID-19 positive mothers in retrieved studies

Type of Placental Abnormalities	Number (n)	Percentage of abnormalities to Total number of abnormal placentae	Percentage of abnormalities to Total number of examined placentae
Changes in Placental weight			
Small Placenta	3	4.8	2.6
Large Placenta	1	1.6	0.8
Microscopic Changes			
Delayed maturation of villous tree	1	1.6	0.8
Terminal villi (capillary congestion and focal microchrangiosis)	1	1.6	0.8
Villous agglutination	1	1.6	0.8
Evidence of Thrombotic tendency in Villous apparatus: Multiple organizing intervillous hemorrhage /thrombi/Avascular Villi/ Fibrosis	40	64	35
Chronic intervillositis	3	4.8	2.6
Funisitis	1	1.6	0.8
Infiltration with Inflammatory cells	4	6.4	3.5
Defective placental barrier	7	11.4	6.2
Placental Swab positive for SARS CoV-2	17	27.4	15
Total number of Placentae found with abnormalities	62		55
Total Number of examined placentae	112		

Table 5 shows the laboratory abnormalities in COVID-19-positive neonates; the commonest laboratory abnormality in affected neonates is lymphopenia encountered in 20% of cases.

**Table 5:** Laboratory abnormalities in COVID-19-positive neonates

	Number	Percentage
No. of neonates positive for COVID-19	45	100
Leukocytosis	6	13.3
Leucopenia	5	11.1
Neutrophilia	3	6.7
Lymphopenia	9	20.0
Reticulocytosis	1	2.2
CRP	2	4.4
Elevated prothrombin time	3	6.7
Elevated ferritin	1	2.2
Elevated AST	4	8.9
Elevated ALT	2	4.4
Elevated bilirubin total	3	7.7
Elevated indirect bilirubin	1	2.2
Elevated IL-6	3	6.7
Elevated IL-10	1	2.2

*ALT* alanine aminotransferase, *AST* aspartate aminotransferase, *COVID-19* coronavirus disease 2019, *CRP* C-reactive protein, *IL* interleukin

**Fig. 2:**
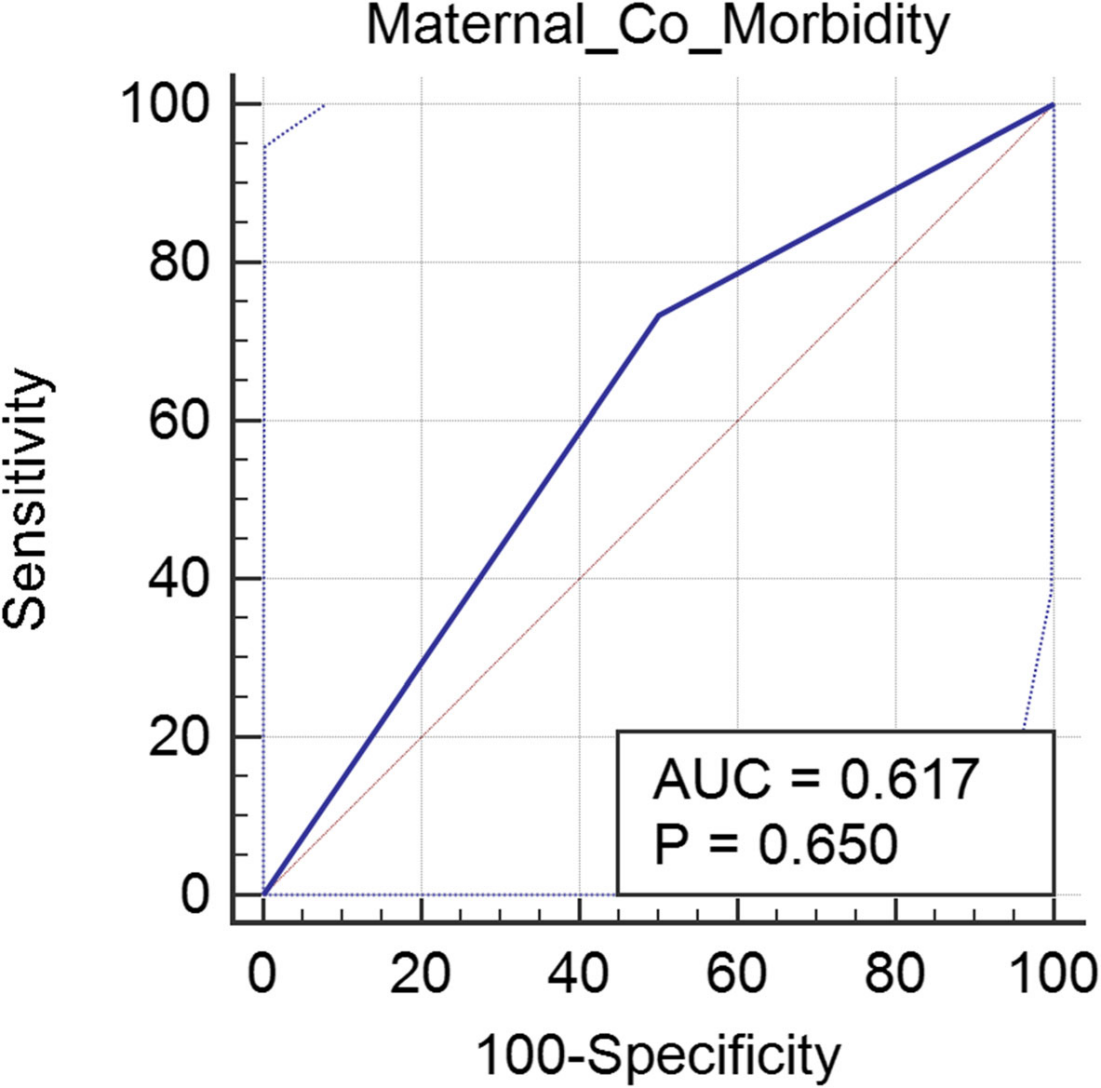
ROC curve showing the relationship between maternal co-morbidities and placental vascular compromise. Abbreviations: AUC: area under the curve, P: Pearson coefficient for statistical significance, ROC: receiver operating characteristics

Figure 2 is a ROC curve proving the absence of relationship between maternal co-morbidities and the occurrence of placental vascular compromise of thrombotic process.

**Fig. 3:**
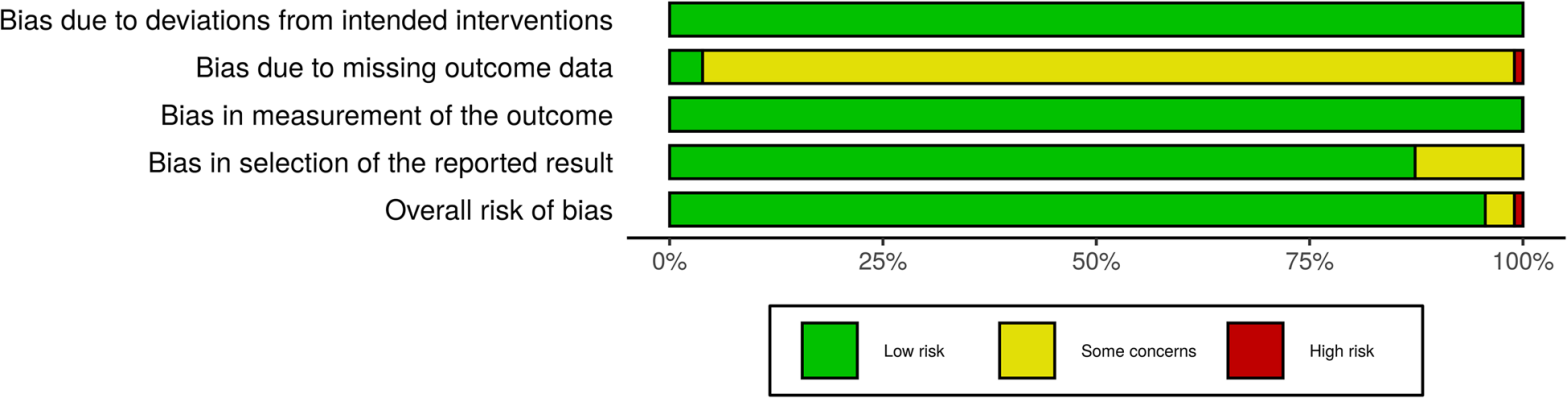
Risk of bias assessment in the included studies

Bias assessment was performed using the Cochrane revised tool for bias assessment and illustrated [77] in Fig. 3; the main defect encountered was the lack of unified outcome parameters in the collected studies. Only 18 studies explored placental abnormalities, with only 72 placentae examined in 1787 COVID-19-positive mothers.

## Discussion

Vertical transmission of COVID-19 follows the same pattern of uncertainty as almost everything concerning COVID-19. New evidences being unraveled every day make meta-analysis the only possible solution to reach consensus about points of dilemma.

This report is by far the largest systematic review to be implemented in this context, not only regarding the number of mother-infant pairs, but also the targeted outcome parameters. The largest report preceding us is Lopes de Sousa et al.’s report [78]. Lopes de Sousa report included 755 pregnancies, while our review studied the outcome of 1787 pregnancies, also Lopes de Sousa report did not focus on placental abnormalities and its correlations with neonatal outcomes.

Our study confirmed the previous impression from the former outbreaks by CoV that transplacental transmission is very unlikely occurring in 2.8% of all positive mothers. Despite being unlikely, it has been reported to happen and this should warrant further studies on the mechanisms underlying the variability of vertical transmission from pregnancy to another.

The commonest laboratory finding in affected neonates was lymphopenia. This finding goes in agreement with the same pattern of haematologic abnormalities encountered in adult patients. The programmed cell death receptor 1 secreted from macrophages in the lung environment as well as from resident T cells leads finally to T cell exhaustion with subsequent lymphopenia in affected patients.

The most intriguing finding uncovered in our review is the strong evidence pointing towards placental damage with subsequent intrauterine hypoxia of the fetus. This finding was supported at several stages in our study. As mentioned earlier, 20% of all infants in whom manifestations have been reported have showed evidence of intrauterine hypoxia. Placental damage could not be attributed to maternal co-morbidities as proven by the ROC analysis performed which showed that maternal co-morbidities failed to predict the occurrence of placental vascular compromise with an insignificant *P* value of 0.6. Moreover, 7 cases were born premature in Shanes et al. series [52], all of which were demonstrating evidence of thrombosis in their respective placentae, with negative swabs for SARS-CoV-2. The timing of swab performance was not clearly mentioned in his analysis. The remaining nine neonates were born full term, 3 of them only showed placental abnormalities; among these 3 neonates, who showed placental abnormalities, two displayed evidence of intrauterine hypoxia and were small for dates. The prevalence of placental abnormalities in premature deliveries and the results of ROC analysis might not be enough to prove the role of COVID-19 in inducing placental damage, but they fortify such hypothesis. More solid findings need to be achieved through case/control studies.

A report by Wang and colleagues [79] suggested that viremia is reported to occur in less than 1% of cases. However, this finding seems to hugely underestimate the burden of viremia in Coronaviridae infections. An old report by Chen et al. [80] during the first SARS outbreak showed that RNA of SARS-CoV can be detected in up to 50% of blood samples and can last up to 1 week.

This old evidence of longstanding viremia might explain the observed placental damage, as placenta is a heavily vascularized organ; however, more studies should correlate the degree of placental damage with the duration and degree of viremia.

Placental changes were more prevalent than COVID-19-positive neonates, 62 vs. 45 respectively, out of which 64% showed evidence of ischemia. Placental changes encountered seemed to mirror the timeline at which infection was detected in COVID-19-positive mothers. Three percent of mothers were infected in the 1^st^ trimester, while defective proliferation and formation of villi was observed in a similar percentage of cases.

Defective formation of villi can be accounted due to the role played by an intracellular enzyme termed Furin in the genesis of placental villi [81–83]. AbdelMassih outlined the important interplay between Furin, COVID-19, and the vascular endothelium, an important constituent of the human placenta [11].

The findings of our study also go in agreement with that of Cardenas and colleagues; Cardenas et al proved that viruses that do not exhibit vertical transmission might cause placental damage. They also proved that viral infection of the placenta can elicit a fetal inflammatory response that, in turn, can cause organ damage and potentially downstream developmental anomalies. Furthermore, we demonstrate that viral infection of the placenta may sensitize the pregnant mother to bacterial products and promote preterm labor [84].

One of the final reports included in our review concluded that vertical transmission is unlikely in COVID-19. The case/control study performed by Edlow and colleagues showed that placental malperfusion even without gross visible pathology in the placenta is not uncommon event, with resultant risk of fetal distress [73].

In view of the above findings, proper hydration and prophylactic anticoagulation might be needed for COVID-19 pregnant women, especially those whose tests suggest strong prothrombotic tendency such as elevated D-Dimer, or those whose abdominal ultrasound and fetal cardiotocography offer a strong evidence of placental insufficiency. The guidelines of several obstetrics and gynecological international societies were clustered by D’Souza et al. and were in agreement with our suggestions [85].

Fetal hypoxia can also impact neonatal outcome. Meconium staining with subsequent risk of meconium aspiration is particularly prevalent in pregnancies complicated with fetal hypoxia. Anticipation of meconium staining in pregnancies complicated by COVID-19 would be highly indicated to neonatologists in the delivery room [86]. Moreover, fetal hypoxia increases the likelihood of persistent pulmonary hypertension and failure of ductal closure after birth, this impact should be considered during postnatal assessment of neonates born to COVID-19 mothers [87].

### Limitations of our study

The sampling time was not reported in 31% of cases which is a non-negligible number putting a huge risk of reporting bias. Forty two percent of positive newborns were tested in the first 12 h after delivery while the remainder 58% of cases were tested after 12 h, raising suspicion of possible postnatal infection.

The lack of homogenous outcome parameter illustrated in Fig. 3 can lead to an underestimation of placental abnormalities; however, the high percentage of placental abnormalities out of the few examined placentae partially resolves this issue. Another limitation of the studies is the relative weight of case reports compared to case series and retrospective studies. Two studies by Kayem et al. [55] and Knight Dphil et al. [53] constitute 58% of all counted in pregnancy outcomes. The limitation of sample size was clearly and extensively discussed by Lopes de Sousa et al. [78].

## Conclusion

The aggregated data in this systematic review are by far the largest to date regarding neonatal outcomes of COVID-19. Results suggest that vertical transmission of COVID-19 is unlikely as it occurred in 2.8% of neonates but underlines an important and underestimated risk, which is the possible placental insufficiency due to the prothrombotic tendency created by COVID-19. These findings should warrant more case/control studies to compare placental abnormalities with the duration and degree of viremia. Also thorough antenatal care should be offered to COVID-19-positive mothers to evaluate their prothrombotic tendency and to monitor their need for anticoagulation. Finally, yet importantly, complications such as meconium aspiration and PPHN should be compared in COVID-19-positive vs. COVID-19-negative mothers and should be anticipated by neonatologists in the delivery room and during follow-up after delivery.

## Data Availability

Not applicable.

## Abbreviations

ACE2: Angiotensin converting enzyme 2
CNS: Central nervous system
COVID-19: Coronavirus disease 19
GA: Gestational age
GERD: Gastro-esophageal reflux disease
GIT: Gastro-intestinal system
HKCoV: Hong Kong Coronavirus
IUGR: Intrauterine growth retardation
MERS: Middle East Respiratory Syndrome
*N*: Number
NEC: Necrotizing enterocolitis
Ob/Gyn: Obstetrics and gynecology
PRISMA: Preferred Reporting Items for Systematic Reviews and Meta-Analysis
RDS: Respiratory distress syndrome
SARS-1: Severe acute respiratory syndrome
